# Dasatinib modulates sensitivity to pemetrexed in malignant pleural mesothelioma cell lines

**DOI:** 10.18632/oncotarget.10428

**Published:** 2016-07-06

**Authors:** Valentina Monica, Marco Lo Iacono, Enrico Bracco, Simone Busso, Laura Di Blasio, Luca Primo, Barbara Peracino, Mauro Papotti, Giorgio Scagliotti

**Affiliations:** ^1^ Department of Oncology, University of Torino, San Luigi Hospital, Orbassano, Torino, Italy; ^2^ Department of Oncology, University of Torino, IRCCS Candiolo, Torino, Italy; ^3^ Department of Clinical and Biological Sciences, University of Torino, San Luigi Hospital, Orbassano, Torino, Italy

**Keywords:** pemetrexed, dasatinib, malignant pleural mesothelioma, cell lines, thymidylate synthase

## Abstract

**Background:**

Thymidylate synthase (TS), one of the key enzymes for thymidine synthesis, is a target of pemetrexed (PEM), a key agent for the systemic therapy of malignant pleural mesothelioma (MPM) and its overexpression has been correlated to PEM-resistance. In MPM, experimental data report activation of the c-SRC tyrosine kinase suggesting it as a potential target to be further investigated.

**Results:**

MPM cell lines showed different sensitivity, being MSTO the most and REN the least sensitive to PEM. REN cells showed high levels of both TS and SRC: dasatinib inhibited SRC activation and suppressed TS protein expression, starting from 100 nM dose, blocking the PEM-induced up regulation of TS protein levels. Dasatinib treatment impaired cells migration, and both sequential and co-administration with PEM significantly increased apoptosis. Dasatinib pretreatment improved sensitivity to PEM, downregulated TS promoter activity and, in association with PEM, modulated the downstream PI3K-Akt-mTOR signaling.

**Cell lines and Methods:**

In three MPM cell lines (MPP89, REN and MSTO), the effects of c-SRC inhibition, in correlation with TS expression and PEM sensitivity, were evaluated. PEM and dasatinib, a SRC inhibitor, were administered as single agents, in combination or sequentially. Cell viability, apoptosis and migration, as well as TS expression and SRC activation have been assessed.

**Conclusions:**

These data indicate that dasatinib sensitizes mesothelioma cells to PEM through TS down-regulation.

## INTRODUCTION

Malignant pleural mesothelioma (MPM) is a highly aggressive tumor strongly linked to asbestos exposure [1, 2]. MPM mortality rates are estimated to increase by 5–10% per year in most industrialized countries, with an expected peak over the period 2020–2050 [3]. In advanced disease, chemotherapy as single agent (PEM) or in doublet combinations (PEM, cisplatin), despite inducing tumor shrinkage and improving overall survival, has mainly a palliative role with a median survival in advanced disease ranging between 9 and 17 months [4].

Intrinsic and acquired resistance mechanisms, including high thymidylate synthase (TS) expression, may be responsible for the limited efficacy of PEM in MPM [5] and, in several cell lines, the induction of resistance to PEM was associated with increased levels of TS mRNA expression [6]. A phase III study reported an inferior activity in terms of overall survival of the combination of cisplatin and PEM compared to cisplatin and gemcitabine for squamous cell lung carcinoma and opposite data for non-squamous non-small cell lung cancer [7] and this differential efficacy was related to TS mRNA expression, being higher in squamous cell carcinoma of the lung [8, 9].

Other anticancer agents may potentially modulate PEM anti-proliferative activity. In pancreatic carcinoma cells the inhibition of SRC, a Src Family Kinase (SFK) member strongly activated in many solid tumors and controlling key processes in tumor development, was shown to revert the 5-FU resistance of cancer cells through indirect TS regulation [10]. SFKs are activated in MPM cell lines and tissue specimens when compared with normal mesothelial cells [11]. In MPM cell lines, SRC inhibitors led to apoptosis, cell cycle arrest and decreased migration and invasion [12] In NSCLC cell lines, the synergistic effect of PEM in combination with dasatinib, an oral dual Bcr/Abl and SFKs competitive ATP inhibitor, has already been reported [13].

The present study investigated whether SRC inhibition may increase, or restore, the sensitivity to PEM in MPM cell lines. This is the first study in MPM cell lines reporting the benefit of a SRC-inhibitor in suppressing the increased TS expression that occurs as a compensatory response to PEM.

## RESULTS

### Characterization of TS expression/localization and SRC activation in MPM cell lines

Malignant pleural mesothelioma (MPM) histology includes three phenotypes: epithelioid, sarcomatoid or biphasic (combination of the two histotypes). The sarcomatoid histotype is the least common (5% of the patients), immortalized sarcomatoid cell lines are very difficult to obtain, and some of those commercially available lack clear-cut distinct features. The MPM cell lines we analyzed include the two most commonly documented histotypes: epithelioid (REN, MPP89) and biphasic (MSTO).

**Figure 1:**
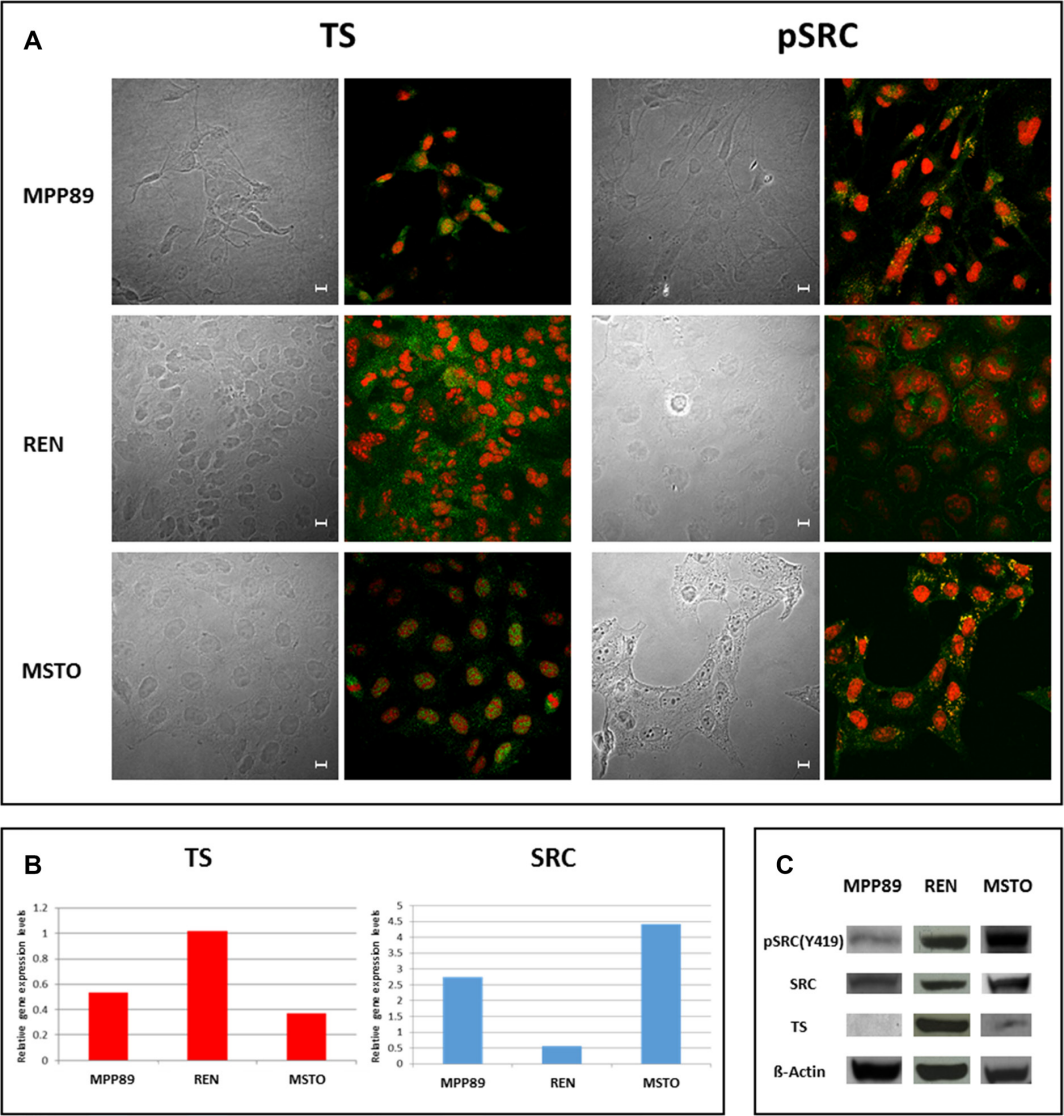
Basal TS, pSRC and SRC localization and expression in MPM cells In all MPM cell lines analyzed, the subcellular distribution of both TS and pSRC was detected by confocal microscopy after immunostaining with anti-TS (FITC-green, left panel) and anti-pSRC (FITC-green, right panel). Propidium iodide was used to counter stain nuclei, and the images were overlaid to determine both TS and pSRC localization within the cell. Confocal series images were taken on an inverted Zeiss LSM510 microscope equipped with a Plan-Apochromat 63X/1.4 oil immersion Ph 3 objective (Oberkochen, Germany). Scale bars: 10 μm. (**A**). Basal TS and SRC genes expression was determined by means of quantitative Real Time PCR (**B**) and TS, SRC and pSRC protein expression was also detected using immunoblotting (**C**). BACT served as housekeeping control.

Expression and localization of TS and both total and activated (phospho Tyr 419, pSRC) SRC baseline levels were investigated in untreated MPM cells. Differences in both TS and pSRC expression and localization were detected: immunofluorescence showed a strong expression of pSRC at the cell membrane (SRC Tyr419, green) and differential TS nuclear and/or cytoplasmic expression (TS, green) in the considered cell lines. TS expression was homogeneously detected in the cytoplasm of all cell lines, extended to the nucleus in MSTO and MPP89. Conversely, pSRC was differentially detected being localized in the cytoplasm of MSTO cells and almost exclusively in the nucleus of MPP89 cells. In REN cells, pSRC was detected in both the cytoplasm (including the cell membrane) and the nucleus (Figure 1A). Baseline SRC and TS expression were also assessed at transcriptional (Figure 1B) and protein levels (Figure 1C): TS gene expression did not show evident differences between MPP89 and MSTO cell lines, in contrast REN expressed about double amount of TS mRNA. TS protein levels correlated with the mRNA levels, being high in REN and scanty in both MPP89 and MSTO. This correlation mRNA-protein was not retained for SRC expression: SRC gene and protein expression levels were not associated and SRC protein, both total and phosphorylated forms, was higher in REN and MSTO compared to MPP89.

### Pharmacological interaction between dasatinib and PEM

**Figure 2:**
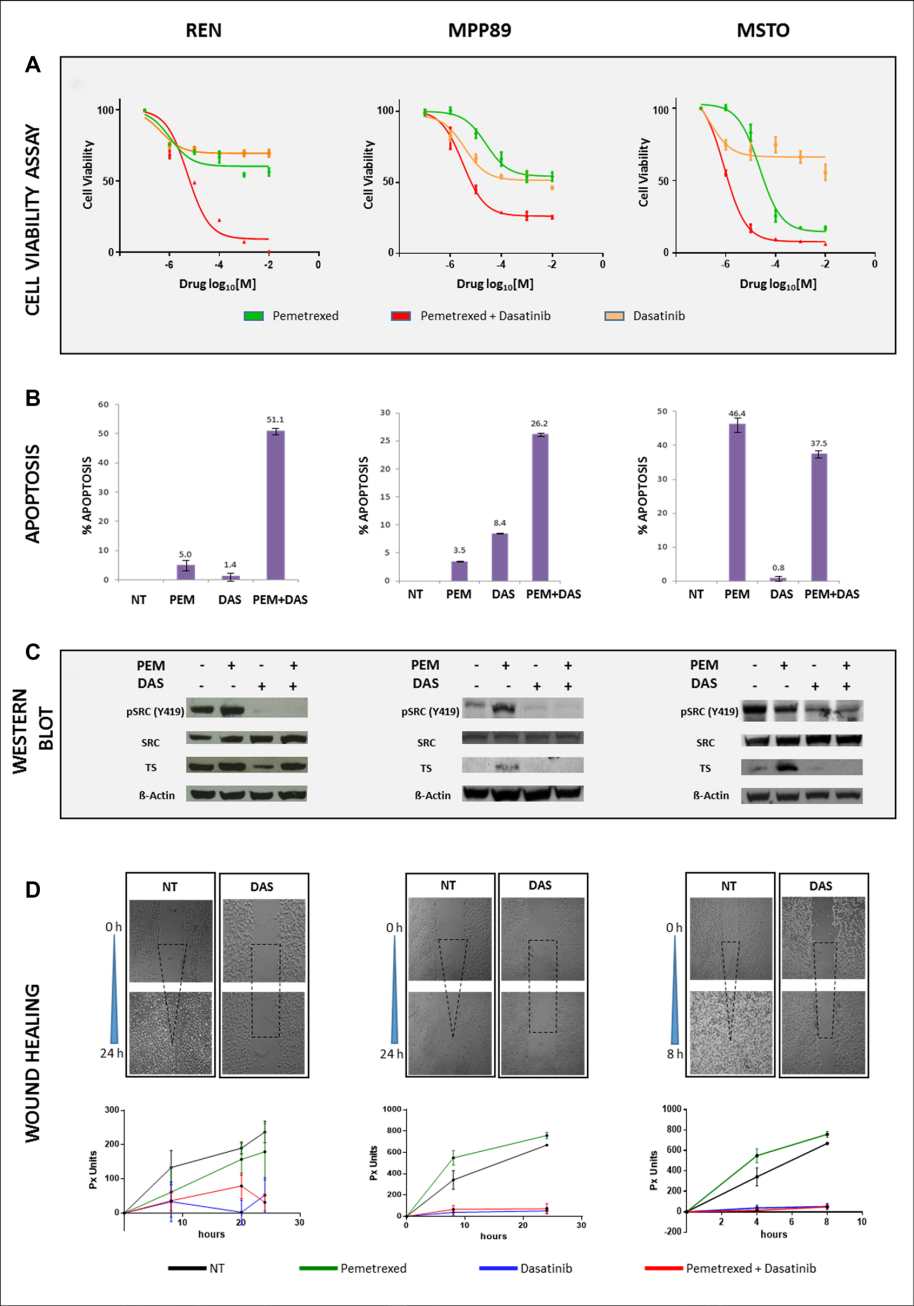
PEM and Dasatinib affect cell viability, apoptosis, TS and SRC protein levels and cell migration PEM and dasatinib efficacy was established in all three MPM cell lines. REN and MPP89 (left and middle panels) were incubated with single and combination drugs for 72 hours, while 48 hours were sufficient for MSTO (right panel) (**A**). The apoptotic rate displayed differential responses, among MPM cells, to single and double treatments after 72 (left and middle graph) and 48 (right graph) hours, respectively (**B**). The same incubation times were applied to detect TS, SRC and pSRC protein expression (**C**). Wound healing assay was employed to determine the effect mediated by drugs on cell migration. Compared with untreated, dasatinib treatment abolished cell migration capability to heal the scrape after 24 hours in both REN and MPP89 cell lines (left and middle panels), and after 8 hours in MSTO (right panel). Plots display the progressive time-depending wound healing, in pixel units (px), under different drugs conditions (**D**).

The effect of PEM and dasatinib on the viability of MPM cells was investigated. PEM weakly affected cell viability in MPP89 and REN cells compared to untreated, while MSTO cells showed the strongest sensitivity, even at lower PEM concentrations. Because of the documented PEM activity, in this cell line experiments lasted after 48 hours. According to the approach reported in the Material and Method section, the best-fit estimate of the IC_50_ for PEM, when combined with dasatinib, was 5 μM in REN cells, 1 μM in MPP89 cells and 0.5 μM in MSTO cells. The effect of dasatinib was more homogeneous, being IC_50_ values 5 μM in REN and 1 μM in both MPP89 and MSTO cells. Dose-response curves are shown in Figure 2A. In REN and MPP89 cell lines, dasatinib shifted the cytostatic activity of PEM to cytotoxic/anti-proliferative effect. In PEM-sensitive MSTO cell lines, the addition of dasatinib significantly reduced cell viability as indicated by the lower IC_50_ values (Figure 2A).

The different efficacy of dasatinib and PEM observed in cell viability experiments, when administered either as single or combined agents, prompted the investigation on apoptosis. After PEM administration, Annexin V-Propidium Iodide staining showed a significant increase of apoptosis in MSTO cells at 48 hours, while a weaker effect was detected in REN and MPP89 at 72 hours (Figure 2B). Conversely, the proportion of REN and MPP89 cell lines undergoing apoptosis was significantly higher than after single agent administration, suggesting a synergistic effect of these drugs. In contrast, the combined treatment in MSTO cells resulted in a reduced apoptotic rate (Figure 2B). These results suggest that even the less PEM-sensitive MPM cell lines, such as REN and MPP89, can be sensitized by the co-administration of dasatinib.

### SRC and TS protein expression and chemosensitivity correlation

In all MPM cells, dasatinib, alone and in combination with PEM, reduced SRC phosphorylation in the activating Tyr 419 (Y419) without affecting total SRC expression levels. PEM differently increased TS expression levels, either alone or in combination with dasatinib. In REN cells, dasatinib as single agent significantly reduced TS protein expression when compared to untreated cells. This suggests a crosstalk between TS and SRC pathways and, more specifically, dasatinib modulates TS expression (Figure 2C).

### Dasatinib, but not PEM, inhibits MPM cell migration

Since dasatinib induced MPM cell line morphological alterations, including cell size reduction and frequently round shape (data not shown), we investigated its effect on cell motility and for this purpose the wound-healing assay was used. Untreated MPM cells were highly motile, being MSTO cells able to repopulate the scratched area within eight hours, while REN and MPP89 cells repaired the wound within 24–30 hours. In presence of dasatinib, cell migration into the wounded area was greatly impaired (Figure 2D). Because of the lack of information on PEM ability to interfere with cell motility, we investigated whether PEM may affect the migratory ability of MPM cells. Cells were treated with IC_50_ doses of single agents or of their combination: PEM did not affect cell migration and, in combination with dasatinib, the migratory inhibition was comparable with that of dasatinib alone. These data suggest that PEM acts synergistically with dasatinib on proliferation rate and in inducing apoptosis, but does not affect, neither promoting nor interfering, with the migratory potential.

### Dasatinib enhances PEM-induced cytotoxicity down-modulating TS expression

**Figure 3:**
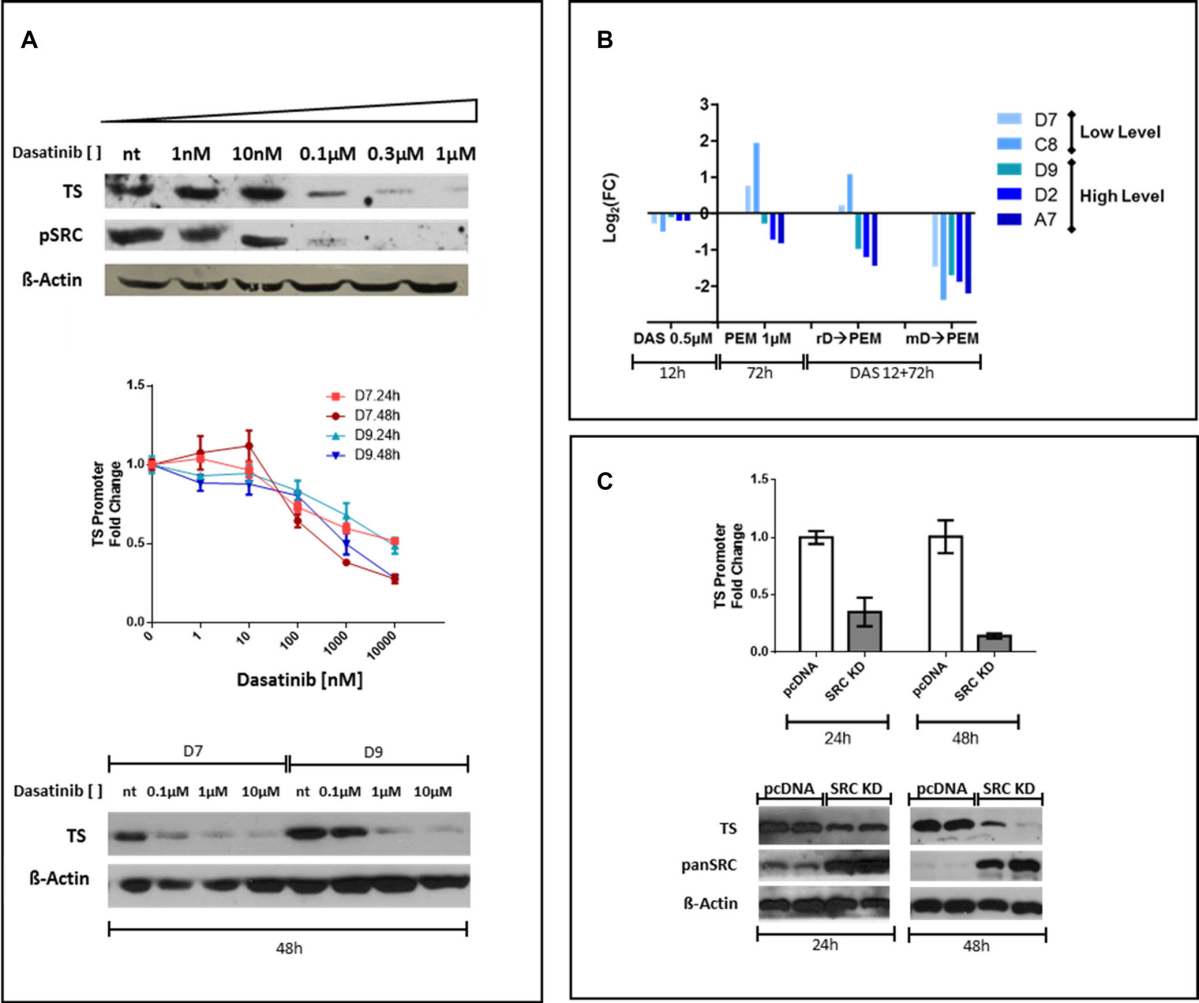
Dose-depending efficacy of dasatinib on TS promoter inhibition and SRC direct involvement in TS promoter/protein modulation REN cells treatment with scalar dasatinib concentration inhibited TS expression and SRC activation already at 100 nM concentration. A similar behavior was seen for the TS-promoter (REN TS-Prom-1191 + 176 clones D7 and D9). In particular, in each clone, dasatinib reduced TS promoter mediated luciferase activity at 24 and 48 h. This effect was also confirmed at the TS protein level (**A**). To assess the effect of combination of dasatinib and PEM we measured the luciferase expression in five independent REN TS- Prom-1191 + 176 clones. After pretreatment for 12 hours with 0.5 μM dasatinib, all clones expressed low levels of luciferase compared to controls. Clones were subsequently incubated for 72 hours with PEM at 1 μM (PEM) either removing (rD→PEM) or maintaining (mD→PEM) dasatinib pretreatment. An incubation with 72 hours of PEM alone was in parallel carried out. In all the clones examined, mD→PEM condition was the most efficient in downregulating TS transcriptional activity (**B**). The expression of a dominant negative form of SRC (SRC K295M) impaired expression of luciferase under control of TS-promoter at both 24 and at 48 h post-transfection. Furthermore, the effect of SRC KD K295M was assessed also at the protein level, resulting in a reduction of the endogenous TS protein (**C**).

Unexpectedly, in REN cells dasatinib at IC_50_ reduced TS expression (Figure 2C, left panel) and we investigated if this effect was SRC-inhibition related. To this purpose, REN were treated for 72 hours with increasing concentrations of dasatinib (1 nM to 1 μM). The lowest dose that completely eliminated SRC phosphorylation (100 nM) was also corresponding to the concentration that down-regulated TS expression (Figure 3A) and TS inhibition was dasatinib concentration dependent (Figure 3A, upper panel). To assess dasatinib role at the transcriptional level, we isolated the very proximal promoter region of TS promoter (−1191+176 bp) and cloned in front of the luciferase reporter gene. Stable REN cell line clones (REN TS-Prom-1191+176) were selected and luciferase activity evaluated after treatment with dasatinib. The experiments on two independent REN TS-Prom-1191+176 clones, D7 and D9, showed that dasatinib impaired luciferase activity both at 24 and at 48 h (Figure 3A, middle panel), mirroring the endogenous TS protein expression pattern (Figure 3A, bottom panel). Furthermore, to evaluate the effect of dasatinib and PEM combination, the luciferase expression in five different REN TS-Prom-1191 + 176 clones that differ among each other because of their basal luciferase expression levels, was measured. In particular, clones D7 and C8 expressed low luciferase levels (< 10000 CPS), in contrast, D9, D2 and A7 that expressed higher luciferase levels (> 30000 CPS). After clones pretreatment for 12 hours with 0.5 μM dasatinib, all of them lowered the luciferase activity in comparison with controls (Figure 3B). Afterwards, the cells were sequentially incubated for 72 hours with 1 μM PEM (PEM), removing (rD→PEM) or maintaining (mD→PEM) dasatinib (D) pretreatment. A further PEM alone administration (72 hours), without dasatinib pretreatment, was also performed. mD→PEM was the most valuable treatment in impairing TS transcriptional activity. Indeed, in all five clones we observed a significant reduction (> 75–80%) of luciferase levels compared to untreated condition. A different pattern was observed after PEM addition, alone or upon dasatinib wash out. Those clones with basal promoter activation increased luciferase activity after PEM treatment; conversely, clones with basal high luciferase activity were roughly unaffected by PEM treatment.

These results could be dependent by clone conditions, suggesting that in those clones with low basal TS promoter activation, the inhibition of TS protein by pemetrexed activates a feedback on promoter increasing the expression of luciferase, while in clones with massive basal activation, the system reaches a plateau level and 1 μM pemetrexed cannot increase the expression of luciferase. Thereby in these conditions, we assessed only the signal reduction mediated by dasatinib.

To better understand the specific involvement of SRC in this scenario, we transfected TS-promoter reporter gene, in the presence or absence of SRC dominant negative (K295M, kinase dead), as well as in presence of dasatinib. The expression of SRC dominant negative impaired luciferase activity, under control of TS-promoter, both at 24 and at 48 hours post transfection, indicating direct regulation of SRC on TS promoter (Figure 3C). The effect of SRC dominant negative was evident also at the protein level, reducing endogenous TS protein (Figure 3C). These results, independent by dasatinib use, clearly demonstrate that SRC controls TS expression through transcriptional regulation.

### Different concentrations of both drugs modifies apoptosis and SRC/TS mRNA expression

Dasatinib effect on TS expression suggests a role as a sensitizer to PEM treatment. Twenty-four hours after REN cells seeding, single agents and sequential treatments (72 hours *rD* → 72 hours PEM and 72 hours or *mD* → 72 hours PEM) were investigated. Cell viability assays demonstrated that pretreatment with dasatinib significantly decreased PEM IC_50_, being the reduction higher in *mD* and co-administration conditions. Afterwards, we assessed whether pretreatment with dasatinib enhanced also the apoptotic event induced by PEM. REN cells were incubated with both drugs, as single agent or in combination, concomitantly or sequentially, either at IC_50_ dose for each drug or at the reduced dose of 100 nM for both drugs. The choice of this concentration has been justified in Figure 3A, where dasatinib 100 nM was the lowest concentration that switched off SRC phosphorylation and reducing TS expression.

**Figure 4:**
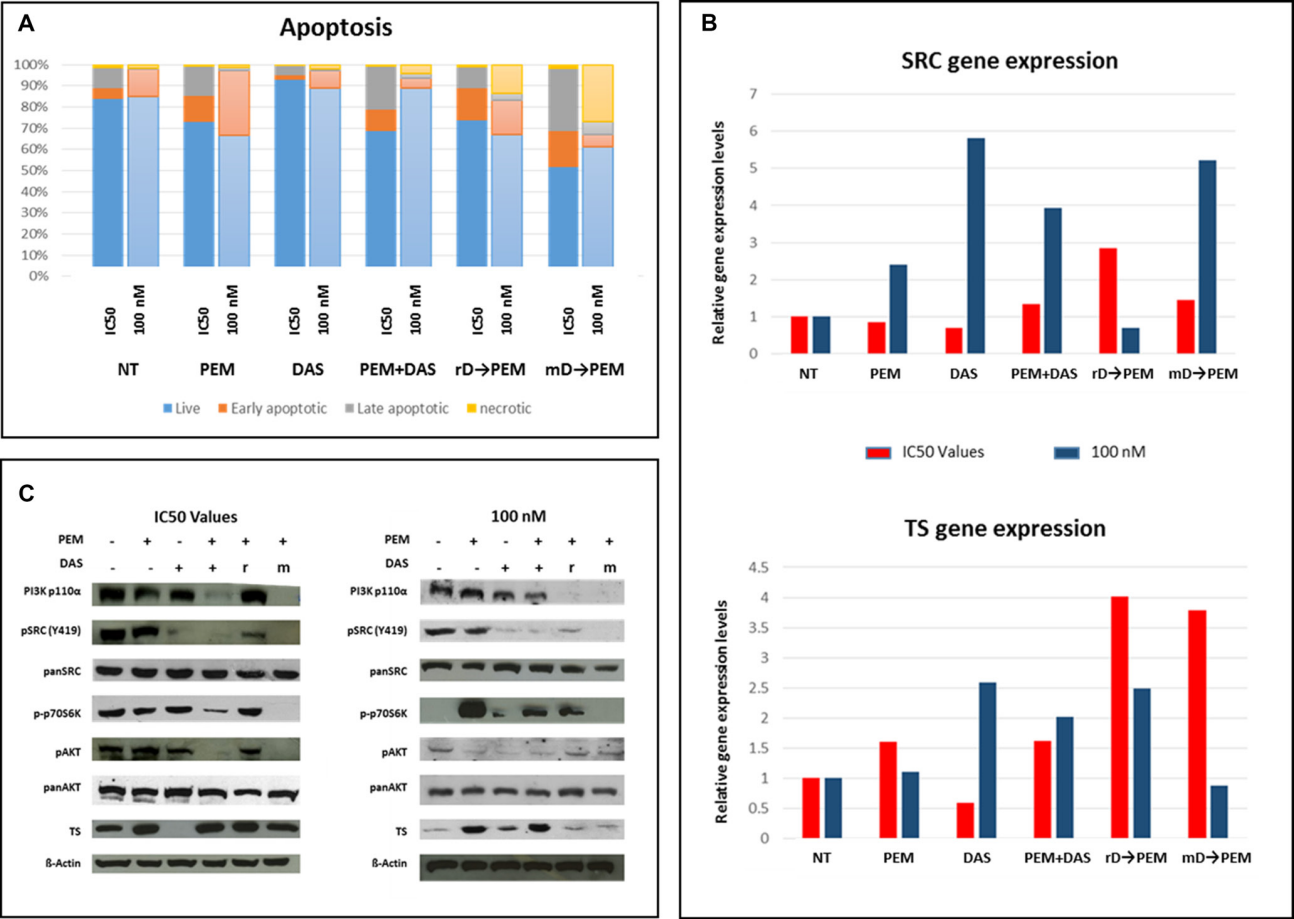
Dasatinib pretreatment effects on apoptosis, target expression and PI3K-Akt-mTOR pathway Upon single agents (PEM and dasatinib), combination and sequential treatments characterized by both, removal (rD→PEM) and maintained (mD→PEM), dasatinib pretreatment apoptosis rate was evaluated. Single and combined treatments were performed for 72 hours, sequential administrations at 72 (dasatinib pretreatment) + 72 hours (PEM incubation). The data are the results of three independent biological experiments for each concentration setting (IC_50_ & 100 nM) (**A**). TS and SRC genes expression was assessed for all the conditions tested in the study: IC_50_ (red) and 100 nM (blue). While TS gene was upregulated when sequential treatments with IC_50_ doses of both drugs were employed, the modulation of SRC gene expression more pronounced at 100 nM dose (**B**). Both, combined and mD→PEM treatments at IC_50_ values downregulated the expression and the activation of the PI3K-AKT-mTOR signaling players analysed. Noteworthy, none of the conditions assayed was able to reduce TS expression levels. On the contrary sequential treatments, at 100 nM, led to TS downregulation (r = rD; m = mD) (**C**).

In REN cells treated with IC_50_ dasatinib, apoptosis was not affected, while treatment with PEM at IC_50_ increased the apoptotic rate by 11.8% when compared to untreated cells. Comparable rates were observed with the sequence *rD* (apoptotic rate 10.3%), and a higher rate with the combination of PEM and dasatinib (16%). The highest apoptotic effect was observed after sequential *m*D (32.4%; Figure 4A). At 100 nM for both drugs, cell death rate was similar: dasatinib alone did not affect apoptosis, while PEM alone or the sequence *rD*, both generated an 18% apoptotic rate. Differently from the results observed testing IC_50_ doses, the simultaneous administration of PEM and dasatinib did not affect cell apoptosis, whereas the sequential *mD* induced a 24% of cell apoptosis (Figure 4A). These data indicate that the sequential administration *mD* led to higher efficacy, also when lower concentrations of the two drugs were tested.

In parallel, TS and SRC mRNA expression were assessed. The most relevant TS gene expression change was observed in dasatinib pre-treated cells (with either removal or maintenance of dasatinib at the time of PEM addition) with a three-fold increase in TS gene expression level compared to untreated cells (Figure 4B, lower panel). At the 100 nM dose, TS expression levels did not significantly change after PEM administration, with a 1.5 fold up-regulation after dasatinib alone or after the sequence *rD*, and an even down-regulating effect in *mD* sequence (Figure 4B, lower panel). SRC expression did not change when drugs were added at IC_50_, showing only an almost two-fold increase in *rD* sequence. At 100 nM dose, SRC expression was strongly (up to five-fold) up-regulated in dasatinib alone treated cells, three-fold with the concomitant administration of dasatinib and PEM and had a four-fold increase with the sequence *mD* (Figure 4B, upper panel).

### Dasatinib and PEM, at low doses, impair intracellular signaling of PI3K-Akt-mTOR pathways

Apoptosis and gene expression data analyses indicate that TS transcriptional regulation is under the control of an upstream tyrosine kinase, such as SRC. Because the differential sensitivity was not related to the histological subtype of investigated cell lines, we tested putative differences in mutational pattern through NGS sequencing of fifty tumor-associated genes. REN cell line showed the loss of the tumor suppressor LKB1/STK11, an intracellular kinase that converges with PI3K signaling to regulate a number of downstream effectors, including the mTORC1 complex. LKB1/STK11 is involved in SRC signaling [14], as well as in PEM response through mTOR pathway [15]. In REN cells, none of the five amplicons of LKB1 included in the panel was detected, differently from MPP89 and MSTO cells and this finding was supported by both qPCR and Western blot experiments. Neither LKB1 mRNA nor protein was detected (Supplementary Figure 1).

These preliminary observations prompted further investigations on the PI3K-Akt-mTOR pathway, with the hypothesis that SRC and TS modulation could converge in this signaling pathway. Western blot analyses reported in Figure 4C show the expression data obtained after treatment with both drugs at IC_50_ doses (upper panel). TS protein was always strongly overexpressed, with the exception of dasatinib that repressed TS expression. SRC phosphorylation was partially restored in the *rD* condition. The PI3K catalytic subunit α (p110-α) was strongly expressed in untreated cells as well as following single agent dasatinib and PEM. While PI3K-p110-α subunit expression was not affected by increasing doses of dasatinib, even at 1 μM (data not shown), the concomitant administration of dasatinib and PEM and the sequence *mD* resulted in a marked abrogation of p110-α subunit, without changes in the expression of the catalytic subunit β (p110-β, data not shown). A similar expression pattern was also observed for AKT phosphorylation (Ser 473) without changes in the total AKT expression levels. The phosphorylation of p70S6K was also assessed, but no modifications were detected in both single agent and *rD* treatments compared to untreated cells. On the contrary, p70S6K phosphorylation was significantly reduced by the concomitant administration of dasatinib and PEM and eliminated in the *mD* condition (Figure 4C). Since dasatinib induced TS suppression at the dose of 100 nM, protein changes in the PI3K-Akt-mTOR pathway were further investigated. Dasatinib suppressed SRC phosphorylation, decreasing AKT activation and reducing p70S6K phosphorylation as well as TS expression (Figure 4C, right panel) while mTOR was activated, with no changes in the PI3K p110-α expression. The phospho-mTOR content was semi-quantitatively assessed and mTOR phosphorylation was slightly increased following the administration of single agent (PEM, dasatinib) or in the concomitant administration, but decreased in sequential administrations (data not shown). Single agent PEM did not influence SRC phosphorylation, but inhibited AKT phosphorylation and strongly influenced the activation of mTOR–p70S6K pathway, leading to increased TS expression (Figure 4C). A similar pattern alteration was observed with the combination of dasatinib and PEM, along with the expected inhibition of SRC phosphorylation secondary to dasatinib. Conversely, sequential treatments decreased mTOR phosphorylation and PI3K p110-α expression, maintaining AKT phosphorylation and surprisingly TS expression at low levels, similar to those detected in untreated cells (Figure 4C). SRC phosphorylation, as well as p70S6K phosphorylation, was suppressed in mD treatments (Figure 4C, lower panel).

## DISCUSSION

In the present study, the potential enhancement of PEM anti-tumor effect by dasatinib, a multitargeted tyrosine kinase inhibitor, was explored in MPM cell lines. Several studies showed an interaction when cytotoxic agents are administered simultaneously with tyrosine-kinase inhibitors [16]. The potential synergistic effect of dasatinib on PEM sensitivity was investigated in three MPM cell lines showing a differential effect on inhibiting downstream effector signaling and cell viability.

PEM inhibits several multiple folate-dependent enzymes, including thymidylate synthase (TS), and in non-small cell lung cancer PEM had a lower antitumor activity in squamous when compared to non-squamous histotypes [7–9] and this differential activity has been associated to higher levels of intracellular TS in squamous histology [6, 17]. Strategies to enhance antifolate efficacy include the ability of other drugs, among which targeted agents, simultaneously or sequentially administered, to decrease TS expression levels or in reducing its up-regulation. While in MPM there is no evidence of activity of tyrosine kinase inhibitors [18, 19], an interaction between c-SRC inhibitors and antifolate agents has been reported, mostly mediated by TS and c-SRC expression level modulation [10, 11, 13, 20, 21]. High basal TS levels characterize REN cells as well as reduced sensitivity to PEM and loss of LKB1/STK11. LKB1 null tumors are associated with clinical aggressiveness as well as with activation of SRC pathway, leading to the hypothesis that LKB1 loss promotes dependence upon this signaling pathway for cell migration [14]. In our study, REN cells had the most aggressive behavior with high SRC expression: dasatinib inhibited cell migration after wound healing stress, as also demonstrated by others [12], even in the presence of PEM, which did not modify the impairment of dasatinib-mediated cell migration.

Single-agent dasatinib did not show activity in unselected previously treated MPM patients [22], and in our *in vitro* data no single agent activity of dasatinib was detected in all three MPM cell lines but in REN cells it reduced TS protein expression. This modulation was also confirmed by luciferase assays and dasatinib after at least 12 hours down-modulated luciferase activity under control of TS promoter. In addition, the combined administration of PEM and dasatinib, as well as the pretreatment with dasatinib, increased the cellular response to PEM. The level of luciferase in all five TS promoter clones showed how sequential treatments, consisting in 12 hours of maintained dasatinib followed by 72 hours of PEM, were able to almost abrogate TS promoter activity. The co-transfection of TS promoter and SRC KD (K295M) demonstrated that this regulation is mainly mediated by SRC, affecting also TS protein levels. This data suggest a dasatinib regulation, through SRC inhibition, on those transcription factors having responsive elements in the region of TS promoter here adopted. Among these putative transcription factors, Sp1, GABP and LSF were found to be regulated by SRC activation, through ERK signaling [23–25]. Further investigation to establish the main TS promoter-responsive region and corresponding transcription factors involved in this regulation are clearly needed. The activity of dasatinib in inhibiting SRC family members, activating an intracellular signaling that converges on mTOR pathway, has been demonstrated in breast cancer models [26]. These findings agree with previous *in vitro* studies reporting that the pretreatment with rapamycin increases PEM activity reducing TS protein expression [27]. The enhanced efficacy of dasatinib in REN cells could be also related to its LKB1 null phenotype, which influences dTTP metabolism [28] and AMPK-mTOR deregulation [15]. This suggests that the loss of LKB1, reducing AMPK activation, makes cells more sensitive to dasatinib which, in turn, induces cellular stress and downregulates both p70S6K and Akt phosphorylation. Conversely, mTOR phosphorylation is increased, most likely due to missed AMPK activation, in the biological attempt to maintain homeostasis [29]. PEM leads to AMP-analog accumulation, which necessarily requires LKB1 for AMPK activation [15]. In micro-environmental stress conditions (including compromised mTOR downstream signaling activation and lower TS levels), the addition of PEM, upon dasatinib exposure, triggers downregulation of mTOR phosphorylation and PI3K p110-α expression, leading to both protein synthesis deficiency and inhibition of proliferation stimuli.

Src family kinase (SFK) is a group of nine non-receptor tyrosine kinases, including, beyond SRC, three members (Yes, Fyn, and Lyn) previously identified as pivotal players in mesothelioma [30, 31]. Dasatinib reduced SRC phosphorylation without significant changes in other SFK members’ expression, except in REN cells, where it induced also a reduction of Lyn expression (data not shown). Previously reported data in a Fyn-deficient MPM cell line showed a decreased Lyn expression following PP2 administration, a SFK inhibitor, which also affected apoptotic induction [30]. PEM and dasatinib synergistically inhibited cell proliferation in all cell lines, while induction of apoptosis was more evident in REN and MPP89 cells and less in MSTO. According to previous studies in MSTO cells [16], the combination of PEM with vandetanib led to a minimal apoptotic effect compared with PEM alone, most likely due to the low TS expression in this biphasic cell line. In REN cells, the higher apoptotic effect of PEM and dasatinib co-treatment was associated with a more intense cytotoxic effect, including suppression of PI3K p110-α (but not the β isoform) subunit expression, and reduction/suppression of SRC, Akt, p70S6K phosphorylation. Unfortunately, the main effect of this pharmacological stimulation consists in the up-regulation of TS expression levels suggesting that TS increase could be the adaptive cellular response to high dose PEM exposure. Dasatinib at 100 nM inhibited SRC phosphorylation, reduced both TS promoter activity and protein expression; in the sequential *mD* treatment testing PEM at 100 nM dose a lack of the compensating cell response was observed, with no TS up-regulation and with induction of apoptosis. At the tested concentration, dasatinib already acts on Akt phosphorylation, as previously reported [16, 32, 33]. In addition, *mD* condition reduces PI3K-p110-α subunit expression, as well as suppress both mTOR and p70S6K phosphorylation: the dependence of p70S6K phosphorylation from SRC has been previously demonstrated through the ability of the SRC-specific inhibitor SU6656 to reduce p70S6K phosphorylation [34].

In conclusion, our data in MPM cell lines demonstrate that the pretreatment with dasatinib sensitizes PEM activity through SRC inhibition, even at low concentrations of both drugs, impairing cell migration, affecting TS promoter regulation and decreasing TS protein, with consequent increase of PEM sensitivity. Sequential administration of the two agents, also at low doses, enhanced the synergistic effect observed, with inhibition of TS protein up-regulation and increased PEM cytotoxic activity. Based on these results, future *in vivo* studies will be planned and new treatments schedules designed to maximize the efficacy of combo treatment.

## MATERIALS AND METHODS

### Cell culture

Three human MPM cell lines (MSTO-211H, NCI-MPP89, and REN) were used: MSTO-211H (designated MSTO) and MPP89 were purchased from Interlab Cell Line Collection (Genova, Italy), while REN [35] cells were kindly provided by Dr. Moro (University of Novara, Italy) and Dr. Albelda (University of Pennsylvania, Philadelphia, USA). Cells were grown in monolayer cultures in RPMI 1640 containing 10% fetal bovine serum, 2 mM glutamine, and 5% Penicillin/Streptomycin at 37°C humidified atmosphere of 95% air and 5% CO_2_.

### Reagents and drugs

Dasatinib [BMS-354825, Bristol-Myers Squibb] was purchased by LC Laboratories (Woburn, MA, USA) and was prepared as a 10 mmol/L stock solution in DMSO. Pemetrexed [ALIMTA] was kindly provided by Eli Lilly Corporation (Indianapolis, IN, USA) and was dissolved in Hanks’ balanced salt solution as a 60 mmol/L stock solution. Preliminary cell culture tests have shown that DMSO (Volume 0.2% was the maximum amount used to deliver dasatinib) has no effect on cell viability, cell cycle, apoptosis, or signaling (data not shown).

For Western blot analysis the following antibodies were used: cSrc (32G6), pSrc, (Tyr419) (D49G4); phosphoinositide 3-kinase (PI3K) p110α subunit (C73F8), PI3K p110β subunit (C33D4) and phospho-ribosomal protein S6K (p-p70S6K [Thr389]) from Cell Signaling Technology (Danvers, MA, USA); Beta-actin (C4); Vinculin (N-19), pAkt1/2/3 (11E6) and Akt (all from Santa Cruz Biotechnology, Dallas, TX, USA); Thymidylate Synthase (TS) (EPR4545), mammalian target of rapamycin (mTOR (ab2732)), from AbCam (Cambridge, UK). Horseradish peroxidase (HRP)-conjugated donkey-anti-goat IgG was from Santa Cruz Biotechnology, HRP-goat-anti-mouse and HRP-goat anti-rabbit polyclonal IgG were from AbCam. For apoptosis assay, Annexin V- fluorescein isothiocyanate (FITC) was purchased from BD Biosciences (San Jose, CA, USA), PI (propidium iodide) from Life Technologies (Carlsbad, CA, USA); for immunofluorescence, FITC-labelled anti-rabbit secondary antibody from Jackson Immuno Research (West Grove, PA, USA).

### Immunofluorescence microscopy

For immunofluorescence analyses, cells were grown on glass coverslips following the procedure previously described [36], except for fixation method employed in this study, consisting in 4% paraformaldehyde/PBS. Confocal series images were taken on an inverted Zeiss LSM510 microscope equipped with a Plan-Apochromat 63X/1.4 oil immersion Ph 3 objective (Oberkochen, Germany). Scale bars: 10 μm. Each experiment was performed in duplicate.

### Cell viability (MTS assay)

For standard cell culture maintenance, cells were seeded in 96-well plates for 24 hours under the above indicated conditions, then treated with this range of increasing concentrations (10 nM, 100 nM, 1 μM, 10 μM, 100 μM and 1 mM) of PEM (A) or dasatinib (B), either as single agents or in combination (ratio 1:1 for each drug concentration) and incubated for 48 and 72 hours. Additionally, in REN cell line, sequential administration of the two agents was also investigated, as follows: cells were incubated with dasatinib for 72 hours, then dasatinib was washed out (*rD*) and PEM added for additional 72 hours at the increasing concentrations reported above (C); in another set of experiments, dasatinib was maintained (*mD*) in cell culture and PEM added for 72 hours (D). Four technical replicates and three biological replicates for each treatment/cell line were performed.

After exposure of cell lines to drugs, an [3-(4-5-dimethylthiazol-2-yl)-5-(3-carboxymethoxyphenyl)- 2-(4-sulfophenyl)-2H-tetrazolium, inner salt MTS assay (Promega, Madison, USA) was performed according to the manufacturer's instruction. Absorbance was measured at 490 nm with EnSpire Multimode Plate Readers (Perkin Elmer, Waltham, Massachusetts). Due to cytostatic effect of both drugs, when administered as single agents (except in MSTO cell line, where PEM showed cytotoxic effect already at 48 hours), the determination of inhibitory concentration of 50% of cells (IC_50_) was not always possible. On the contrary, when the two drugs were used in combination, the administration produced a cytotoxic behavior, demonstrating strong synergy of the two drugs. For this purpose, in each cell line and for both drugs, we called “PEM IC_50_” and “dasatinib IC_50_” the corresponding drug doses which, in combination, were able to inhibit 50% of cells. These values were obtained by means of the GraphPad software (La Jolla, CA, USA).

### Apoptosis assay

Apoptosis was evaluated in sub-confluent cells treated as reported above and single agent or combinations were added for 48–72 hours. After incubation, cells were harvested (supernatant and adherent cells), washed twice with 1X PBS, re-suspended in 1X Binding solution (100 mM HEPES, pH 7.4; 140 mM NaCl; 2.5 mM CaCl_2)_ and then stained with Annexin V-FITC and PI. The following controls were used to set up compensation and quadrants: unstained cells, cells stained with Annexin V-FITC alone (no PI), cells stained with PI alone (no Annexin V-FITC). After 15 minutes of incubation in the dark, cells were analyzed on a cytofluorimeter by fluorescence activated cell sorting analysis (CyAn™ ADP Analyzer - Beckman Coulter; CA, USA) using Summit 4.3 software.

### Migration assay (wound healing-scratch assay)

MPM cells were grown to confluence on 6-well dishes, and then three separate scrapes for each well were made in the confluent monolayer using a sterile pipette tip. The monolayer was washed with 1X PBS, then complete medium containing the corresponding IC_50_ of dasatinib (IC_50_ values included in Figure 2A), alone or in combination with PEM, was supplied. DMSO alone acted as control. Serial photographs of the same scraped section were captured every 6 hours for 30 hours. The width of scratch, over the margins of the wounds, was measured (in pixel values) at the beginning and at regular intervals of 6 hours and then plotted on a graph, to quantify the cells migration rate. The time required for the scrape to heal, in each cell line, was also recorded.

### RNA extraction and gene expression analysis

RNA was extracted with Trizol (Qiagen) following manufacturer's instructions. Each sample was quantified using spectrophotometer and 1 μg of RNA was retrotranscribed as previously described [36]. Genomic DNA contamination was removed by DNAse I treatment, according to manufacturer's protocol (Ambion, Life Technologies, Carlsbad, CA, USA). Relative cDNA quantification was performed using a fluorescence-based real-time detection method with measurements done in triplicate and the comparative Ct method used, as previously reported [37]. Quantitative Real Time polymerase chain reaction (qPCR) was performed with an ABI PRISM 7900HT Sequence Detection System (Applied Biosystems, Life Technologies) in 384-wells plate and SRC primers and probe used were previously reported [13]. For the other genes, exon-spanning TaqMan Gene Expression Assays were used, including TS (TYMS; Hs00926279_g1) and GUSB (GUSB; Hs99999908_m1). Each qPCR reaction was performed in technical triplicate and reported data represent the median value of three independent biological experiments.

### Western blot and alpha technology analysis

From each cell culture plate, adherent cells were washed with 1X PBS, detached using 1X PBS-0.01% EDTA buffer and collected by centrifugation. The obtained pellet was rinsed with ice-cold 1X PBS and lysed for 30 min on ice, vortexing at 10 minute intervals. Extraction cell-lysis buffer was used (Invitrogen, Life Technologies). 100X Protease Inhibitors Cocktail (P8340, Sigma Aldrich, Saint Louis, MO, USA) and 100X Phosphatase Inhibitor Cocktail 2 (P5726, Sigma Aldrich) were adequately added to lysis buffer. Lysates were spun in a centrifuge at 13,700 rpm for 10 minutes at 4°C and the clear supernatant collected. Bicinchoninic acid (BCA, Sigma Aldrich) assay was used to determine protein concentration in each sample and equal protein aliquots (40 μg) were resolved by SDS-PAGE, transferred to nitrocellulose membranes, immuno-blotted with adequate primary antibodies, detected with horseradish peroxidase–conjugated secondary antibodies and revealed by enzymatic chemiluminescence reagent (Millipore, Merck KGaA, Darmstadt, Germany).

To measure the phosphorylation of endogenous mTOR in cellular lysates, a quantitative assay that employs the bead-based Alpha Technology [AlphaScreen SureFire mTOR (p-Ser2448) assay kit (Perkin Elmer, Waltham, MA, USA)] was used. To this purpose, according to the manufacturer's instructions, also total mTOR protein expression was investigated by western blotting. This allowed normalizing the results obtained on phosphorylated mTOR, comparing the two different expression levels.

### Stable transfections: engineering of REN-derived clones expressing luciferase gene under control of TS promoter

To evaluate the ability of different drugs (Dasatinib and PEM) to modulate TS gene expression, we engineered the pGL4.14[luc2/Hygro] plasmid (Promega) with a genomic fragment extract from TS locus by PCR. The genomic fragment correspond to a region, including the transcription-starting codon ATG, of the TS promoter (from nucleotide – 1191 to +176). This fragment was amplified using primers: TYMS.–1191.FW (AATTGGATCCTAAAACTGGGAAATGTGGTCTATTA AAA) and TYMS.+176.RW (AATTAAGCTTGAATACC GACAGGGTGCCG). The PCR was performed with GoTaq Flexy kit (Promega) in buffer containing: 100 ng REN genomic DNA, Taq Buffer 1x, 1.5 mM MgCl2, 1 μM primers, 1.25 u Taq and 10% DMSO. The purified fragment was cloned in pGEMt-easy (Promega) to create pGEMt-TS-Promoter (pGEMt-TS-Prom) plasmid. From this plasmid was cut the region of TS promoter by BAM-HI/HIND III and subsequently cloned in pGL4.14[luc2/Hygro] linearized with BGL II/HIND III generating plasmid pGL4-TS-Prom-1191 + 176. One day prior to transfection, REN cells (1.5 × 10^5^ cells/well) were seeded into a 6-well tissue culture plate and cultured with 2 mL fresh complete RPMI-1640 medium. Cells were then transfected with DreamFect (OZ Biosciences, SAS, Marseille, France) using 1 μg of pGL4-TS-Prom-1191+176 per well according to manufacturer's instructions. After transfection, cells were cultured for 48 hours and subsequently selected by 600 μg/ml Hygromycin b. After 2 weeks incubation with antibiotic, cells were diluted to single cell clones and cultured in presence of 400 μg/ml Hygromycin b. Several clones expressing luciferase under control of TS promoter (D7, D9, D2, C8, A7) were analyzed by luminometer (Enspire; Perkin Elmer) and were employed to quantify the response of TS promoter to dasatinib, PEM and drugs combination.

### Transient transfections: luciferase assays with TS promoter and SRC dominant negative K295M (KD) constructs

A DNA fragment containing the full length *Gallus Gallus* SRC KD K295M sequence was extracted by XbaI from plasmid SG5-SRC-KD and was cloned in pcDNA-3(−) generating plasmid pcDNA-SRC-KD-K295M. The day before transfection, REN cells (2 × 10^5^ cells/well) were seeded into a 6-well tissue culture plate and cultured with 1.5 mL fresh complete RPMI-1640 medium. Cells were then transfected with JetPrimer (PolyPlus, Strasbourg, France) using 2 μg of plasmid 1:10:1 combinations: pGL4-TS-Prom-1191+176/TK-renilla/pdDNA 3 or pGL4-TS-Prom-1191+176/TK-renilla/pcDNA-SRC-KD-K295M per well according to manufacturer's instructions. After transfection, cells were cultured for 24 or 48 hours. After incubation the cells were lysed by Passive Lysis Buffer (Promega) and luminescence measured by luminometer (Enspire). All experiment was performed at least in duplicate for tree times, each firefly luciferase signal was normalized by renilla luciferase and the normalized values was relativized to pcDNA transfection.

### Next-generation sequencing (NGS)

Genomic DNA (gDNA) was extracted using Wizard^®^ Genomic DNA Purification Kit (Promega) following the manufacturer's instructions. gDNA was quantified using fluorometer Qubit platform (Invitrogen, Carlsbad, CA). NGS analyses were performed on the Ion Torrent Personal Genome Machine (PGM, Life Technologies, Grand Island, USA). Cell lines were tested with a commercial library kit (Ion AmpliSeq Cancer Hotspot Panel v.2, CHP2) and the NGS procedure was previously published [38].

### Statistical analysis

Statistical significance of the observed differences in drug-treated and untreated cells was analyzed fitting data to sigmoidal dose-response curve using GraphPad Prism software version 5.0 c. Unpaired *t*-tests for the differences in log IC_50_ for PEM alone and in combination with dasatinib were significant in all experiments with *p*-value < 0.001.

## SUPPLEMENTARY MATERIALS FIGURE



## References

[1] Robinson BW, Musk AW, Lake RA (2005). Malignant mesothelioma. Lancet.

[2] Tsao AS, Wistuba I, Roth JA, Kindler HL (2009). Malignant pleural mesothelioma. Journal of clinical oncology.

[3] Goswami E, Craven V, Dahlstrom DL, Alexander D, Mowat F (2013). Domestic asbestos exposure: a review of epidemiologic and exposure data. International journal of environmental research and public health.

[4] Grosso F, Scagliotti GV (2012). Systemic treatment of malignant pleural mesothelioma. Future oncology.

[5] Roe OD, Szulkin A, Anderssen E, Flatberg A, Sandeck H, Amundsen T, Erlandsen SE, Dobra K, Sundstrom SH (2012). Molecular resistance fingerprint of pemetrexed and platinum in a long-term survivor of mesothelioma. PloS one.

[6] Buque A, Aresti U, Calvo B, Sh Muhialdin J, Munoz A, Carrera S, Azkona E, Rubio I, Lopez-Vivanco G (2013). Thymidylate synthase expression determines pemetrexed targets and resistance development in tumour cells. PloS one.

[7] Scagliotti GV, Parikh P, von Pawel J, Biesma B, Vansteenkiste J, Manegold C, Serwatowski P, Gatzemeier U, Digumarti R, Zukin M, Lee JS, Mellemgaard A, Park K (2008). Phase III study comparing cisplatin plus gemcitabine with cisplatin plus pemetrexed in chemotherapy-naive patients with advanced-stage non-small-cell lung cancer. Journal of clinical oncology.

[8] Ceppi P, Volante M, Saviozzi S, Rapa I, Novello S, Cambieri A, Lo Iacono M, Cappia S, Papotti M, Scagliotti GV (2006). Squamous cell carcinoma of the lung compared with other histotypes shows higher messenger RNA and protein levels for thymidylate synthase. Cancer.

[9] Monica V, Scagliotti GV, Ceppi P, Righi L, Cambieri A, Lo Iacono M, Saviozzi S, Volante M, Novello S, Papotti M (2009). Differential Thymidylate Synthase Expression in Different Variants of Large-Cell Carcinoma of the Lung. Clinical cancer research.

[10] Ischenko I, Camaj P, Seeliger H, Kleespies A, Guba M, De Toni EN, Schwarz B, Graeb C, Eichhorn ME, Jauch KW, Bruns CJ (2008). Inhibition of Src tyrosine kinase reverts chemoresistance toward 5-fluorouracil in human pancreatic carcinoma cells: an involvement of epidermal growth factor receptor signaling. Oncogene.

[11] Menges CW, Chen Y, Mossman BT, Chernoff J, Yeung AT, Testa JR (2010). A Phosphotyrosine Proteomic Screen Identifies Multiple Tyrosine Kinase Signaling Pathways Aberrantly Activated in Malignant Mesothelioma. Genes Cancer.

[12] Tsao AS, He D, Saigal B, Liu S, Lee JJ, Bakkannagari S, Ordonez NG, Hong WK, Wistuba I, Johnson FM (2007). Inhibition of c-Src expression and activation in malignant pleural mesothelioma tissues leads to apoptosis, cell cycle arrest, and decreased migration and invasion. Molecular cancer therapeutics.

[13] Ceppi P, Rapa I, Lo Iacono M, Righi L, Giorcelli J, Pautasso M, Bille A, Ardissone F, Papotti M, Scagliotti GV (2012). Expression and pharmacological inhibition of thymidylate synthase and Src kinase in nonsmall cell lung cancer. International journal of cancer.

[14] Carretero J, Shimamura T, Rikova K, Jackson AL, Wilkerson MD, Borgman CL, Buttarazzi MS, Sanofsky BA, McNamara KL, Brandstetter KA, Walton ZE, Gu TL, Silva JC (2010). Integrative genomic and proteomic analyses identify targets for Lkb1-deficient metastatic lung tumors. Cancer cell.

[15] Rothbart SB, Racanelli AC, Moran RG (2010). Pemetrexed indirectly activates the metabolic kinase AMPK in human carcinomas. Cancer research.

[16] Giovannetti E, Zucali PA, Assaraf YG, Leon LG, Smid K, Alecci C, Giancola F, Destro A, Gianoncelli L, Lorenzi E, Roncalli M, Santoro A, Peters GJ (2011). Preclinical emergence of vandetanib as a potent antitumour agent in mesothelioma: molecular mechanisms underlying its synergistic interaction with pemetrexed and carboplatin. British journal of cancer.

[17] Takezawa K, Okamoto I, Okamoto W, Takeda M, Sakai K, Tsukioka S, Kuwata K, Yamaguchi H, Nishio K, Nakagawa K (2011). Thymidylate synthase as a determinant of pemetrexed sensitivity in non-small cell lung cancer. British journal of cancer.

[18] Nowak AK, Millward MJ, Creaney J, Francis RJ, Dick IM, Hasani A, van der Schaaf A, Segal A, Musk AW, Byrne MJ (2012). A phase II study of intermittent sunitinib malate as second-line therapy in progressive malignant pleural mesothelioma. Journal of thoracic oncology.

[19] Papa S, Popat S, Shah R, Prevost AT, Lal R, McLennan B, Cane P, Lang-Lazdunski L, Viney Z, Dunn JT, Barrington S, Landau D, Spicer J (2013). Phase 2 study of sorafenib in malignant mesothelioma previously treated with platinum-containing chemotherapy. Journal of thoracic oncology.

[20] Lee KH, Hur HS, Im SA, Lee J, Kim HP, Yoon YK, Han SW, Song SH, Oh DY, Kim TY, Bang YJ (2010). RAD001 shows activity against gastric cancer cells and overcomes 5-FU resistance by downregulating thymidylate synthase. Cancer letters.

[21] Ahn JY, Lee JS, Min HY, Lee HY (2015). Acquired resistance to 5-fluorouracil via HSP90/Src-mediated increase in thymidylate synthase expression in colon cancer. Oncotarget.

[22] Dudek AZ, Pang H, Kratzke RA, Otterson GA, Hodgson L, Vokes EE, Kindler HL, Cancer and Leukemia Group B (2012). Phase II study of dasatinib in patients with previously treated malignant mesothelioma (cancer and leukemia group B 30601): a brief report. Journal of thoracic oncology.

[23] Rudge TL, Johnson LF (2002). Synergistic activation of the TATA-less mouse thymidylate synthase promoter by the Ets transcription factor GABP and Sp1. Experimental cell research.

[24] Dong S, Lester L, Johnson LF (2000). Transcriptional control elements and complex initiation pattern of the TATA-less bidirectional human thymidylate synthase promoter. Journal of cellular biochemistry.

[25] Shapira S, Granot G, Mor-Tzuntz R, Raanani P, Uziel O, Lahav M, Shpilberg O (2012). Second-generation tyrosine kinase inhibitors reduce telomerase activity in K562 cells. Cancer letters.

[26] Yori JL, Lozada KL, Seachrist DD, Mosley JD, Abdul-Karim FW, Booth CN, Flask CA, Keri RA (2014). Combined SFK/mTOR inhibition prevents rapamycin-induced feedback activation of AKT and elicits efficient tumor regression. Cancer research.

[27] Kawabata S, Chiang CT, Tsurutani J, Shiga H, Arwood ML, Komiya T, Gills JJ, Memmott RM, Dennis PA (2014). Rapamycin downregulates thymidylate synthase and potentiates the activity of pemetrexed in non-small cell lung cancer. Oncotarget.

[28] Liu Y, Marks K, Cowley GS, Carretero J, Liu Q, Nieland TJ, Xu C, Cohoon TJ, Gao P, Zhang Y, Chen Z, Altabef AB, Tchaicha JH, Wang X, Choe S, Driggers EM (2013). Metabolic and functional genomic studies identify deoxythymidylate kinase as a target in LKB1-mutant lung cancer. Cancer discovery.

[29] Hall A, Meyle KD, Lange MK, Klima M, Sanderhoff M, Dahl C, Abildgaard C, Thorup K, Moghimi SM, Jensen PB, Bartek J, Guldberg P, Christensen C (2013). Dysfunctional oxidative phosphorylation makes malignant melanoma cells addicted to glycolysis driven by the (V600E)BRAF oncogene. Oncotarget.

[30] Eguchi R, Kubo S, Takeda H, Ohta T, Tabata C, Ogawa H, Nakano T, Fujimori Y (2012). Deficiency of Fyn protein is prerequisite for apoptosis induced by Src family kinase inhibitors in human mesothelioma cells. Carcinogenesis.

[31] Sato A, Sekine M, Virgona N, Ota M, Yano T (2012). Yes is a central mediator of cell growth in malignant mesothelioma cells. Oncology reports.

[32] Chen J, Lan T, Zhang W, Dong L, Kang N, Fu M, Liu B, Liu K, Zhang C, Hou J, Zhan Q (2015). Dasatinib enhances cisplatin sensitivity in human esophageal squamous cell carcinoma (ESCC) cells via suppression of PI3K/AKT and Stat3 pathways. Archives of biochemistry and biophysics.

[33] Chen KC, Yang TY, Wu CC, Cheng CC, Hsu SL, Hung HW, Chen JW, Chang GC (2014). Pemetrexed induces S-phase arrest and apoptosis via a deregulated activation of Akt signaling pathway. PloS one.

[34] Rebholz H, Panasyuk G, Fenton T, Nemazanyy I, Valovka T, Flajolet M, Ronnstrand L, Stephens L, West A, Gout IT (2006). Receptor association and tyrosine phosphorylation of S6 kinases. The FEBS journal.

[35] Smythe WR, Kaiser LR, Hwang HC, Amin KM, Pilewski JM, Eck SJ, Wilson JM, Albelda SM (1994). Successful adenovirus-mediated gene transfer in an *in vivo* model of human malignant mesothelioma. The Annals of thoracic surgery.

[36] Lo Iacono M, Monica V, Vavala T, Gisabella M, Saviozzi S, Bracco E, Novello S, Papotti M, Scagliotti GV (2015). ATF2 contributes to cisplatin resistance in non-small cell lung cancer and celastrol induces cisplatin resensitization through inhibition of JNK/ATF2 pathway. International journal of cancer.

[37] Livak KJ, Schmittgen TD (2001). Analysis of relative gene expression data using real-time quantitative PCR and the 2(-Delta Delta C(T)) Method. Methods.

[38] Lo Iacono M, Monica V, Righi L, Grosso F, Libener R, Vatrano S, Bironzo P, Novello S, Musmeci L, Volante M, Papotti M, Scagliotti GV (2015). Targeted next-generation sequencing of cancer genes in advanced stage malignant pleural mesothelioma: a retrospective study. Journal of thoracic oncology.

