# Supplementary material for: Dasatinib modulates sensitivity to pemetrexed in malignant pleural mesothelioma cell lines
Source: Oncotarget. 2016 Jul 6;7(47):76577–89. doi: 10.18632/oncotarget.10428 (PMC5363531; doi:10.18632/oncotarget.10428)
Supplement: Supplementary file 1 [file oncotarget-07-76577-s001.pdf]

# Dasatinib modulates sensitivity to pemetrexed in malignant pleural mesothelioma cell lines

## Supplementary Materials

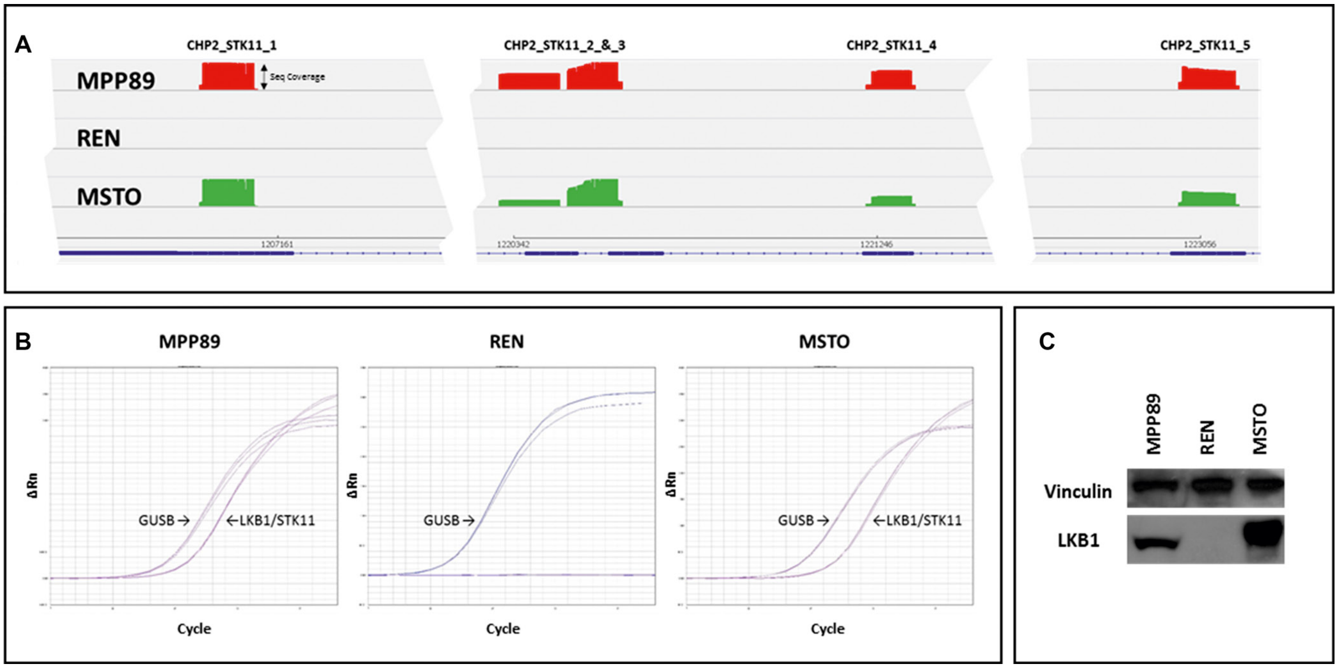


**Supplementary Figure S1: LKB1/STK11 expression in MPM cells.** Next generation sequencing (NGS) characterized LKB1/STK11 status in all MPM cells (**A**): all five amplicons included in CHP2 panel were amplified in both MPP89 (first line) and MSTO (third line), while no detection was available in REN cells (middle line). Real-time PCR (**B**) and western blotting (**C**) show LKB1/STK11 gene and protein expression, respectively, confirming the complete absence of LKB1/STK11 in REN cells.
